# The circulating levels of CTRP1 and CTRP5 are associated with obesity indices and carotid intima-media thickness (cIMT) value in patients with type 2 diabetes: a preliminary study

**DOI:** 10.1186/s13098-021-00631-w

**Published:** 2021-01-26

**Authors:** Ziba Majidi, Solaleh Emamgholipour, Abolfazl Omidifar, Soheil Rahmani Fard, Hossein Poustchi, Mehrnoosh Shanaki

**Affiliations:** 1 Department of Clinical Biochemistry, School of Medicine, Tehran University of Medical Sciences, Tehran, Iran; 2 Department of Medical Laboratory Sciences, School of Allied Medical Sciences, Student Research Committee, Shahid Beheshti University of Medical Sciences, Tehran, Iran; 3 Liver and Pancreatobiliary Diseases Research Center, Digestive Diseases Research Institute, Tehran University of Medical Sciences, Tehran, Iran; 4 Department of Medical Laboratory Sciences, School of Allied Medical Sciences, Shahid Beheshti University of Medical Sciences, Tehran, Iran

**Keywords:** Type 2 diabetes, CTRP1, CTRP5, Carotid intima-media thickness

## Abstract

**Background:**

There is growing evidence that the C1qTNF-related protein (CTRP) family has a crucial role in the pathophysiology of metabolic disorders such as type 2 diabetes (T2D) and obesity. We sought to identify the association of CTRP1 and CTRP5 circulating levels with various obesity parameters such as visceral adipose tissue (VAT) thickness, visceral adiposity index (VAI), and with carotid intima-media thickness (cIMT) in patients with T2D and controls.

**Methods:**

This preliminary study consisted of men with T2D (n = 42) and men without T2D (n = 42). The measurement of cIMT and VAT thickness was performed using an Accuvix XQ ultrasound. Circulating levels of CTRP1, CTRP5, and adiponectin were measured by enzyme-linked immunosorbent assay (ELISA).

**Results:**

CTRP-1 and CTRP1/CTRP5 ratio were markedly higher in patients with T2D compared to controls (p < 0001 and p = 0004 respectively). Interestingly, binominal logistic regression revealed that a higher circulating level of CTRP1 was associated with the presence of T2D (odds ratio [OR]: 1.009 [95% CI: 1.004–1.015]; P = .001). CTRP1 circulating levels were correlated with WHR, VAT, and HOMA-IR in the whole population study. Also, we observed that the ratio of CTRP1 to CTRP5 in plasma (β = 0.648, P = 0.005) and CTRP5 circulating levels (β = 0.444, P = 0.049) are independently associated with cIMT value.

**Conclusions:**

Our results indicated that CTRP1 and CTRP5 concentrations were correlated with atherosclerosis in men with T2D and these adipokines might have a causal role for cardiometabolic risk in T2D.However, more studies in large sample sizes are required to clarify the role of CTRPs in T2D pathogenesis.

## Background

The prevalence of type 2 diabetes (T2D) and associated disorders such as cardiovascular disorders (CVDs) is significantly rising worldwide [1]. As a complex disease, T2D affects the function of the adipose tissue, skeletal muscle, liver, and heart, which implies the possible connection of T2D with multiple metabolic disorders [2–4]. As repeatedly noted, obesity and T2D are closely associated with the incidence of CVDs [5, 6]. Specifically, T2D and CVDs have common risk factors such as obesity and insulin resistance. Moreover, it is generally accepted that CVD is the leading cause of mortality among T2D patients, which results from several abnormalities in cellular metabolism and energy homeostasis. Recent years have seen an intense focus on the understanding of how T2D, obesity, and CVDs are associated together [7].

Adipose tissue plays a major role in the overall metabolism regulation by using secreting a wide range of adipokines. Adiponectin and its paralogues; the family of C1q/TNF-related proteins (CTRPs), seems to be the crucial molecules in the cross-talk among metabolic disorders [8].

The family of CTRPs consists of 15 protein members that play key roles in a variety of metabolic conditions [9, 10]. More importantly, accumulating evidence points toward the possible role of CTRPs in regulating signaling pathways pertinent to glucose and lipid metabolism, insulin signaling, and inflammation [11]. The alteration in circulating levels of CTRPs has been reported in various metabolic diseases such as T2D, obesity, CVD, fatty liver disease, and metabolic syndrome [11–15].

CTRP1 is expressed in various tissues including adipose tissue, liver, muscles, kidneys, and heart [9]. CTRP1 is thought to ameliorate metabolic dysfunction. Specifically, CTRP1 can activate 5′ AMP-activated protein kinase (AMPK) and the mitogen-activated protein kinase (MAPK) signaling pathways, promotes glucose uptake, ameliorates insulin resistance, and increases energy expenditure [16, 17]. The increased level of CTRP1 in T2D, prediabetes, coronary artery disease, congestive heart failure, and atherosclerosis is also reported in several studies [18, 19]. The available evidence, albeit with conflicting results points that CTRP1 regulates inflammatory pathways.

Although there is evidence on the protective role of CTRP1 in murine heart injuries, the exact role of CTRP1 in these conditions is still not fully understood and requires further studies [20]. CTRP5 is another member of the CTRPs family that is expressed with the highest expression in adipose tissue [18]. Mounting evidence points to the role of CTRP 5 in regulating energy metabolism, insulin signaling, inflammatory pathways, migration, and proliferation in vascular smooth muscle cells which all are involved in the pathomechanism of T2D and CVDs.In detail, CTRP5 increases glucose uptake via stimulating incorporation of the glucose transporter 4 (GLUT4) into the plasma membrane by a mechanism dependent on AMPK phosphorylation. Besides, phosphorylation of acetyl-CoA carboxylase (ACC) is mediated by CTRP5 which results in fatty acid oxidation in rat myocytes [9, 21, 22]. CTRP5 can increase transcytosis of LDL via activating 12/15- lipoxygenases (LOX) expression, a crucial enzyme involved in LDL trafficking and oxidation of low-density lipoprotein[23]. Decreased levels of CTRP5 have been observed in human studies in the context of metabolic syndrome, T2D, and coronary artery disease. The association between CTRP5 and metabolic disorders has been studied with contradicting results, either augmenting or ameliorating insulin resistance and atherosclerosis and this has been reviewed in detail elsewhere [11].

Considering a close association between T2D, obesity, and an increased occurrence of CVD risk factors, it is of great importance to detect the possible molecules which link among the aforementioned conditions.

To reach this purpose, we investigated to address the association of circulating CTRP1 and CTRP5 with carotid intima-media thickness (cIMT) as well as various obesity indices including body mass index (BMI), waist, hip, waist-to-hip ratio (WHR), visceral adipose tissue (VAT), and visceral adiposity index (VAI) in patients with T2D.

## Methods

### Study population

This study consisted of T2D patients (n = 42) and control subjects (subjects without T2D) (n = 42). All participants (T2D patients and controls) were men between the ages of 43–72 years. Informed written consent was obtained from all participants before the study, and the study was approved by the Ethics Committee of the Tehran University of Medical Sciences (TUMS).

Patients with T2D were consecutively recruited from the outpatient clinic of Shariati Hospital, affiliated with Tehran University of Medical Sciences, Tehran, Iran from March 2012 until November 2013. All patients in this study were clinically definite diagnosed with T2D based on the T2DM, the basis of American Diabetes Association (ADA) criteria which were fasting blood glucose (FBG) ≥ 126 mg/dl (7.0 mmol/l) or 2 h plasma glucose ≥ 200 mg/dl (11.1 mmol/l) during an oral glucose tolerance test (OGTT) or random plasma glucose ≥ 200 mg/dl (11.1 mmol/l) [24]. T2D Patients with evidence of any (1) chronic or acute systemic diseases such as infectious disease, (2) acute or chronic renal failure, (3) malignancies, (4) congenital cardiac disease, and (5) type 1 diabetes (T1D) were excluded.

Subjects without T2D as the control group were selected from the same geographical areas and were selected among subjects attending the Shariati Hospital, Tehran, Iran for a routine check-up. The exclusion criteria for controls were as follows (1) T2D; (2) T1D; (3) chronic or acute systemic diseases such as infectious disease; (4) acute or chronic renal failure; (5) malignancies, and (6) congenital cardiac disease. It should be noted that none of the participants were current smokers and alcohol drinkers. All controls were received a regular medical check-up by a physician. Regarding the history of receiving medication, the current use of antidiabetic drugs was reported in 8 patients with T2D. Moreover, 10 patients with T2D and 2 individuals in controls were receiving antihypertensive drugs. We would like to stress that this study is reported in compliance with STROBE guidelines (Supplementary material).

### Ultrasound methods

Ultrasound examinations for measurement of the cIMT and visceral adipose tissue thickness (VAT) were performed using an Accuvix XQ ultrasound unit (Medison, Seoul, Korea) equipped with a 3–7 MHz curved-array and a 5–12 MHz linear-array transducer. The technique for measuring cIMT and VAT has been previously described [25, 26]. In brief, cIMT measured at its thickest point on the distal wall of the carotid arteries, along a 1.5–2 cm proximal to the carotid bulb. cIMT on the left and right sides was evaluated and mean values of both sides were determined as carotid IMT. Also, VAT (in millimeter) was measured as the distance between the anterior wall of the aorta and the internal face of the rectus abdominis muscle perpendicular to the aorta.

### Anthropometric and clinical characterization

Anthropometric indices of including age, weight, height, BMI, WC, hip, WHR, and blood pressure were examined. BMI was measured based on the ratio of weight in kg divided by height in m^2^ to assess participants' obesity. WC using a flexible inch strip in the middle between the lowest rib and the iliac crest was calculated. Furthermore, the hip was measured at the maximum circumference of the buttocks. WHR was measured based on the ratio of WC in centimeters divided by hip circumference in centimeters. After a 15-min rest in a sitting position, systolic and diastolic blood pressures were measured by a manual sphygmomanometer. VAI, as a gender-specific mathematical index was calculated based on simple anthropometric [BMI and WC] and metabolic [TG and HDL Cholesterol (HDL)] parameters [27].

Males: VAI = $$(\frac{WC}{39.68+(1.88 \times BMI)}$$) $$\times$$ $$(\frac{TG}{1.03})$$ $$\times$$ $$\frac{1.31}{HDL}$$

### Measurement of biochemical and laboratory parameters

Fresh venous blood samples were collected into sterile tubes containing the EDTA-K2 after overnight fasting for measuring biochemical analyses. Fasting blood glucose (FBG), urea, creatinine, TG, total cholesterol (TC), low-density lipoprotein cholesterol (LDL-C), HDL-C, aspartate aminotransferase (AST), alanine aminotransferase (ALT), alkaline phosphatase (ALP), and gamma-glutamyl transferase (γ-GT) were measured by autoanalyzer using commercial kits (Pars Azmoon, Tehran, Iran). Additionally, fasting plasma insulin was calculated by enzyme-linked immunosorbent assay (ELISA) kit (Monobind Inc., USA). To examine the IR, homeostasis model assessment of IR (HOMA-IR) was calculated with the equation of fasting blood glucose (mg/dL) × fasting blood insulin (µU/mL) / 405.

### Measurement of plasma level of adiponectin

Plasma levels of adiponectin were determined by using the ELISA Kit (Elabscience, Wuhan, China) according to the manufacturer’s protocol. Intra-assay and inter-assay Coefficients of Variability (CV) were < 10%.

### Measurement of plasma levels of CTRP5 and CTRP1

CTRP1 concentration was measured by ELISA kit (Biovendor research and diagnostic products) with a minimum detectable concentration of 0.016 ng/ml. Intra assay Coefficients of Variability (CV) was 2.7% and inter-assay CV was 8.5%. Plasma levels of CTRP5 were measured by immunoassay using the Cayman system kit according to the manufacturer’s protocol. The inter-assay variability and intra-assay variabilities were 6.975 and 6.3%, respectively.

### Bias

Selection bias was addressed by closely matching cases to controls based on age. Moreover, all participants were men.

### Statistical analysis

The sample size was calculated based on our previous studies. In detail, we estimated that considering alpha = 0.05 and power = 90%, a difference of 50% in the mean value of CTRP5 circulating levels between the two studied groups could be detected with a minimum of 30 subjects in each group. Here, we included 42 subjects in each group.

Continuous variables with normal distribution were presented as mean ± standard deviation (SD) and variables with non-normal distribution were presented as median (interquartile ranges (IQR)). Descriptive analysis was applied and normality was tested for all quantitative variables using the Shapiro–Wilk test. The student’s t-test and the Mann–Whitney U test were used to compare continuous variables between two groups for data with normal and non-normal distribution, respectively. Pearson's correlation coefficient was used to determine the association of CTRP1, CTRP5, and CTRP1/ CTRP5 levels with anthropometric indices and biochemical parameters. It should be mentioned that variables with non-parametrical distribution were log-transformed before analysis. Moreover, linear regression modeling was used to assess the association between the association of CTRP1, CTRP5, and CTRP1/ CTRP5 levels with obesity indices and cIMT, adjusted for history of receiving medication. It should be noted that the linear regression model when we found a significant correlation between CTRP1, CTRP5, and CTRP1/ CTRP5 levels with obesity indices and cIMT.

A p-value < 0.05 was applied to interpret all achieved data from analysis. All data analysis was performed using SPSS 20 (SPSS, Chicago, IL, USA).

## Results

### Anthropometric, biochemical, and clinical characteristics of the study population

Anthropometric, clinical, and laboratory data of T2D patients and control subjects are shown in Table 1. All subjects were men and there is no statistical difference between the two studied groups in terms of age (P = 0.622). However, the T2D group had increased values of WC and WHR compared to controls (P = 0.039 and P = 0.012, respectively). However, other obesity parameters including hip, VAT, and VAI were not comparable between the two groups. We should be noted that BMI was higher in T2D in comparison with the control group but did not reach our threshold of statistically significant difference.

**Table1:** Anthropometric and laboratory characteristics of the study population

Characteristics	Controls (n = 42)	T2DM (n = 42)	P value
Age, years	51 (48–57.25)	55 (46.50–59.25)	0.622
Waist, cm	99.5 ± 10.2	104. 5 ± 11.3	0.039
Hips, cm	102.4 ± 6.4	103.8 ± 7.2	0.363
WHR, -	0.97 ± 0.05	1.004 ± 0.06	0.012
Height, cm	169.5 ± 5.7	168.1 ± 5.7	0.281
Weight, kg	78.0 ± 11.9	81.3 ± 12.1	0.212
BMI, kg/m2	27.13 ± 3.72	28.78 ± 4.33	0.064
FBG, mg/dL	93.50 (87.01–99.17)	146 (123.45–183.07)	0.000
Insulin, μU/mL	8 (3.50–10.10)	7.10 (4.35–9.55)	0.906
HOMA-IR, -	1.79 (0.83–2.43)	2.61 (1.58–3.77)	0.009
Triglycerides, mg/dL	129.10 (93.85–160.30)	142.30 (108.28–184.40)	0.214
Cholesterol, mg/dL	196.50 (167.85–213.90)	202.60 (167.88–221.45)	0.545
HDL, mg/dl	50.10 (44.45–55.90)	55.90 (44.23–64.25)	0.225
LDL, mg/dL	113.71 ± 32.23	115.73 ± 37.56	0.796
LDL to HDL, -	2.26 ± 0.67	2.152 ± 0.63	0.451
Urea, mg/dL	30.46 ± 7.58	32.20 ± 6.55	0.272
Creatinine, mg/dL	1.27 ± 0.18	1.21 ± 0.18	0.164
AST, U/L	18.70 (16.20–23.93)	20.20 (15.98–26.15)	0.679
ALT, U/L	20.50 (14.30–29.65)	22.80 (14.98–40.20)	0.255
ALP, U/L	227 (196.50–263)	220 (185.75–285)	0.810
γ-GT, U/L	24.33 (19.58–32.10)	28.34 (21.69–43.91)	0.045
SBP, mmHg	122 (113.75–140)	132 (120–150)	0.081
DBP, mmHg	80(70–90)	80 (74.25–90)	0.876
Visceral Fat, %	60.76 ± 22.35	66.26 ± 21.90	0.258
WBC, × 10^9^/L	5.40 (1.9)	6.70 (2)	0.029
cIMT, mm	0.79 ± 0.10	0.83 ± 0.12	0.086
VAI	1.60 (1.11–2.05)	1.67 (1.25–2.27)	0.577

Continuous variables with normal distribution were described as mean ± SD and with non-normal distribution were described as Median (IQR)

*T2D* type 2 diabetes, *WC* waist circumference, *WHR*waist-to-hip ratio, *BMI* body mass index, *FBG* fasting blood glucose, *HOMA-IR* homeostasis model assessment of insulin resistance, *TG* triglycerides, *TC* total cholesterol, *HDL-C* high-density lipoprotein cholesterol, *LDL-C* low-density lipoprotein cholesterol, *ALT* alanine aminotransferase, *AST* aspartate aminotransferase, *ɤ-GT* gamma-glutamyl transferase, *ALP* alkaline phosphatase, *SBP* systolic blood pressure, *DBP* diastolic blood pressure, *VAI* visceral adiposity index, *WBC* white blood cells, *cIMT* carotid intima-media thickness

As expected, patients with T2D had higher FBG concentration, insulin levels, and HOMA-IR in comparison with controls. The concentration of TG, HDL-C, LDL- C showed no significant difference between patients and controls.

All liver function-related tests including AST and ALT with expect to GGT showed higher levels in the T2D group compared to controls. Moreover, cIMT as a measurement of subclinical atherosclerosis was higher in T2D patients compared to controls but did not reach our threshold of statistically significant difference (P = 0.086).

### Circulating levels of CTRP5, CTRP1, adiponectin in patients with T2D and controls

**Fig. 1:**
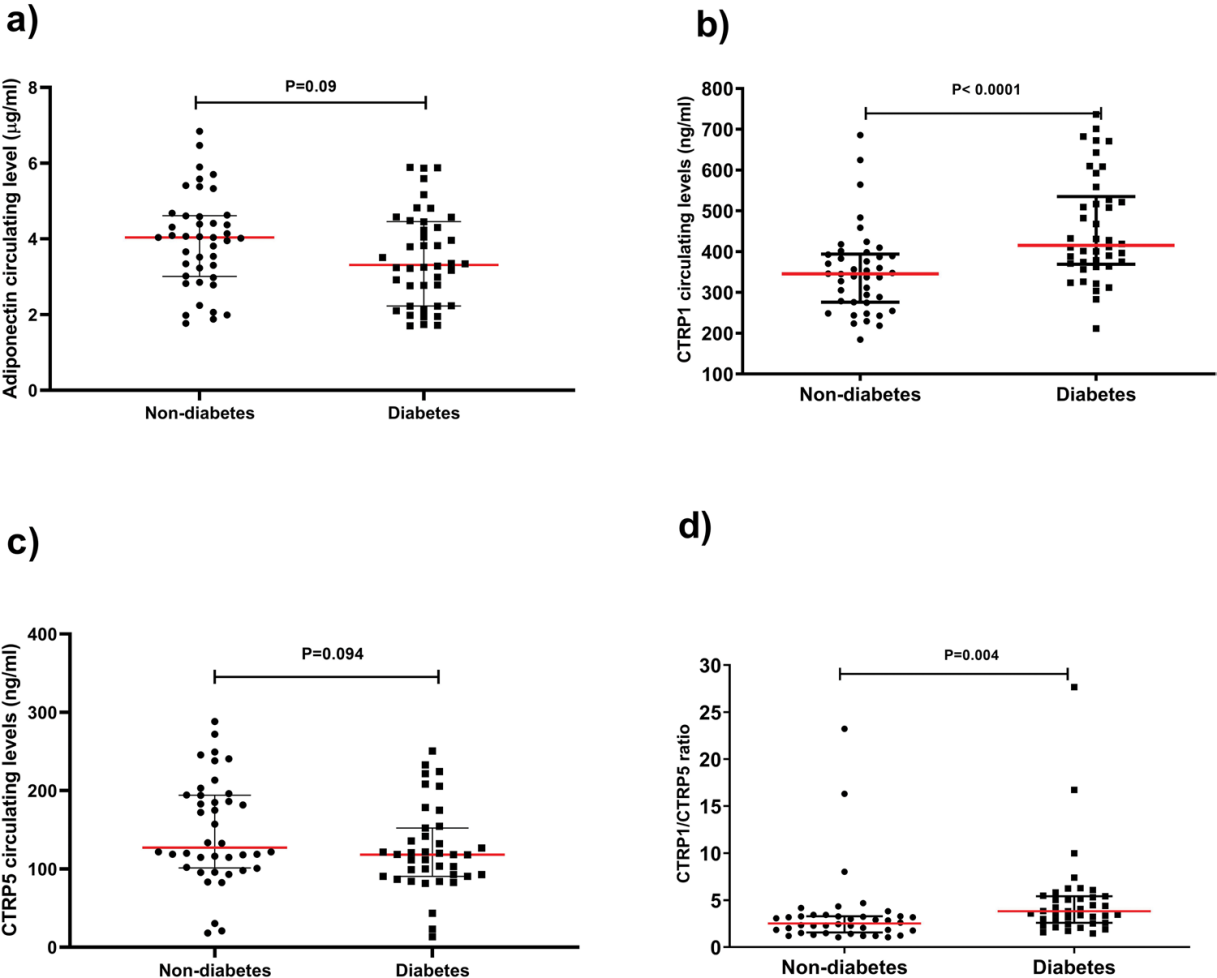
Plasma concentrations of adiponectin (**a**), CTRP1 (**b**), CTRP5 (**c**), and CTRP1/CTRP5 ratio (**d**) in non-T2D and T2D groups. The data are presented as median (Interquartile range). T2D: Type 2 diabetes. Independent Student’s t-test on logarithmically transformed data was used to determine the differences of the adipokines between the two groups

The comparison of CTRP5, CTRP1, adiponectin circulating levels, and ratio of CTRP1 to CTRP 5 between T2D patients and controls **(**Fig. 1**)** revealed that CTRP-1 and CTRP1/CTRP5 ratio were significantly higher in patients with T2D rather than in controls (P < 0001 and P = 0004 respectively). While, plasma levels of both CTRP5 and adiponectin with a borderline significance, was lower in T2D patients in comparison with controls (P = 0.09 and P = 0.094 respectively).

### The association of circulating levels of CTRP1, CTRP5, and adiponectin with T2D

We also performed binomial logistic regression to investigate whether the ratio of CTRP1 to CTRP5 and plasma levels of CTRP1, CTRP5, and adiponectin might predict the presence of T2D (Table 2). When all afore-mentioned items were inserted, we found the one unit increase in the circulating level of CTRP1 was associated with the presence of T2D (odds ratio [OR]: 1.009 [95% CI: 1.004–1.015]; P = 0.001). After adjustment for BMI, an increase in circulating levels of CTRP1 remained a significant risk factor of T2D (odds ratio [OR]: 1.009 [95% CI: 1.004–1.015]; P = 0.001). To identify independent predictors of CTRP1 circulating levels, we performed multivariate stepwise linear regression analysis with age, BMI, WC, hip, WHR, VAI, and VAT as independent variables. Our results showed that WHR (β = 0.273, P = 0.014) is the only predictor for CTRP1 concentration in all participants.

**Table 2:** Binomial logistic regression for an odds ratio of T2D according to the ratio of CTRP1 to CTRP5 and plasma levels of CTRP1, CTRP5, and adiponectin (Model 1) and the ratio of CTRP1 to CTRP5 and plasma levels of CTRP1, CTRP5, and adiponectin (Model 2)

	B	S.E	Wald	P-value	OR	95% CI. for EXP(B)	
						Lower	Upper
Model 1							
CTRP1	0.009	0.003	11.432	0.001	1.009	1.004	1.015
CTRP5	− 0.008	0.006	2.052	0.152	0.992	0.980	1.003
Adiponectin	− 0.268	0.228	1.383	0.240	0.765	0.489	1.196
CTRP1/CTRP5	− 0.015	0.075	0.039	0.843	0.985	0.851	1.141
Model 2							
CTRP1	0.009	0.003	10.679	0.001	1.009	1.004	1.015
CTRP5	− 0.008	0.006	1.758	0.185	0.992	0.981	1.004
Adiponectin	− 0.231	0.233	0.987	0.321	0.793	0.503	1.253
CTRP1/CTRP5	− 0.008	0.076	0.012	0.911	0.992	0.855	1.150
BMI	0.051	0.065	0.610	0.435	1.052	0.926	1.196

*CI* confidence interval, *S.E.* standard error of the mean, *OR* odds ratio

### Correlation of CTRP1/CTRP5, CTRP1 and CTRP5 levels with anthropometric, clinical, and biochemical variables

**Fig. 2:**
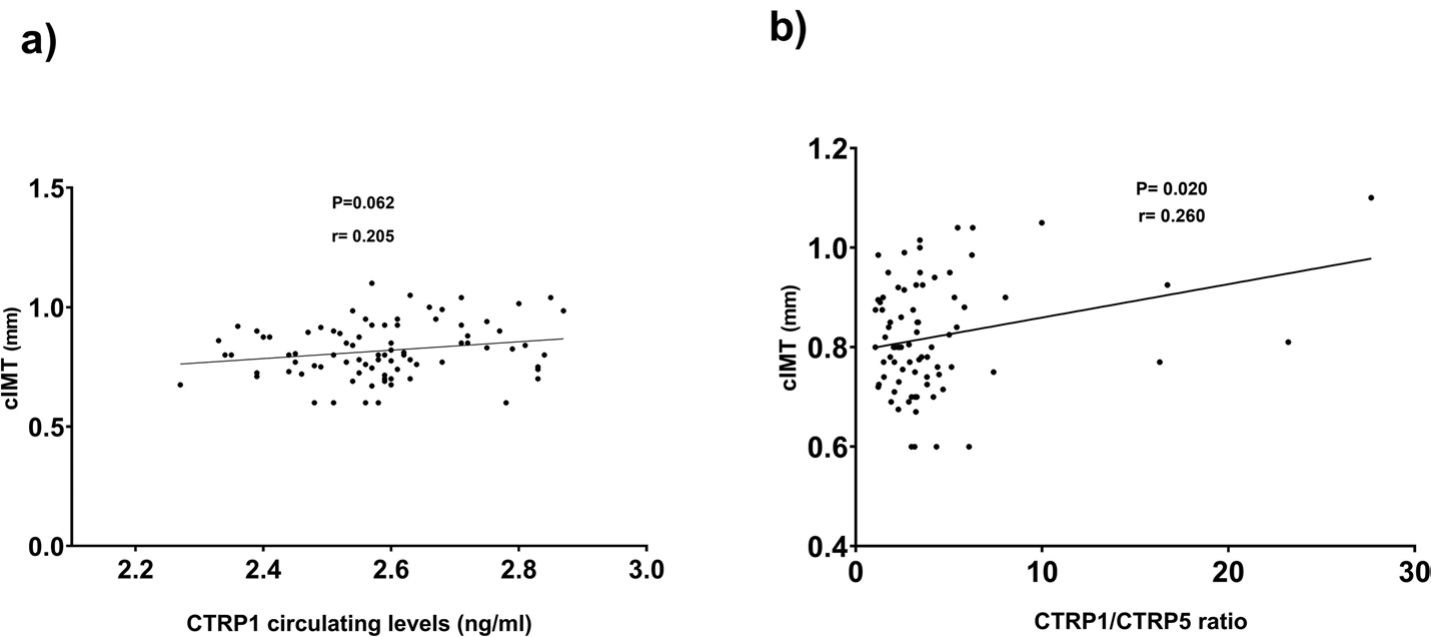
Linear regression plot regarding the correlation of (**a**) CTRP1 circulating levels and (**b**) CTRP1/CTRP5 ratio with cIMT in the whole population. R coefficients and p-value were displayed. Data for CTRP1 circulating levels were logarithmically transformed

The results of the correlation analysis of CTRP1/CTRP5 and circulating levels of CTRP1 and CTRP5 with anthropometric and biochemical characteristics in all participants, T2D patients, and controls were depicted in Tables 3 and 4. We found a positive significant correlation between CTRP1 levels with WHR and HOMA-IR in the whole population study. Moreover, there was a correlation between CTRP1 circulating levels with cIMT (P = 0.062) (Fig. 2a), VAI (P = 0.093), and VAT (P = 0.051). Multiple linear regression models adjusted for history of receiving medication showed that serum CTRP1 levels were significantly associated with WHR (r = 0.244, p = 0.021), and HOMA-IR (r = 0.257, p = 0.017). Moreover, CTRP5 circulating levels inversely correlated with the HOMA-IR index (p = 0.008). Multiple linear regression models adjusted for history of receiving medication showed that serum CTRP5 levels were inversely associated with HOMA-IR (r = -0.239, p = 0.033). Furthermore, the ratio of CTRP1 to CTRP5 positively correlated with cIMT (P = 0.020) (Fig. 2b) and HOMA-IR (P = 0.015) in all participants. Multiple linear regression models adjusted for history of receiving medication showed that serum CTRP1 levels were significantly associated with HOMA-IR (r = 0.239, p = 0.034), but not related to Cimt (r = 0.206, p = 0.078).

**Table 3:** The correlation of CTRP1 and 5 circulating levels with anthropometric characteristics and biochemical data in T2D and controls

	Diabetes		Non-diabetes		Diabetes		Non-diabetes		Diabetes		Non-diabetes	
	CTRP 1,ng/mL				CTRP 5, ng/mL				CTRP 1/5			
	r	p	r	p	r	p	r	p	r	p	r	p
CTRP 1, ng/mL	1		0.017	0.916	0.147	0.371	0.017		0.094	0.568	0.196	0.025
CTRP 5, ng/mL	0.147	0.371	0.196	0.225	1	0.000	1	0.916	− 0.886	0.000	− 0.865	0.000
CTRP 1/5,-	0.094	0.568	0.209	0.430	− 0.886	0.650	− 0.865	0.000	1	0.772	1	
Adiponectin, μg/mL	− 0.039	0.806	− 0.125	0.184	− 0.216	0.186	− 0.085	0.602	0.231	0.157	0.162	0.319
Age, years	− 0.073	0.646	0.209	0.900	− 0.075	0.650	0.128	0.433	0.048	0.772	0.049	0.762
SBP, mmHg	− 0.215	0.171	0.020	0.384	− 0.097	0.557	0.145	0.371	− 0.006	0.970	− 0.081	0.621
DBP, mmHg	0.047	0.769	0.138	0.715	− 0.072	0.664	0.020	0.900	0.073	0.660	− 0.071	0.662
VAI, −	0.213	0.186	0.142	0.249	0.007	0.968	− 0.287	0.081	− 0.08	0.637	0.262	0.112
BMI, kg/m^2^	0.047	0.767	0.058	0.003	0.263	0.106	− 0.192	0.234	− 0.284	0.080	0.166	0.306
WHR, −	0.182	0.249	0.182	0.205	0.260	0.110	− 0.134	0.409	− 0.232	0.156	0.148	0.364
HOMA-IR, −	− 0.001	0.996	0.443	0.003	− 0.292	0.072	− 0.267	0.096	0.225	0.169	0.274	0.087
cIMT, mm	0.091	0.569	0.200	0.205	− 0.396	0.013	0.211	0.192	0.453	0.004	− 0.045	0.781
Visceral Fat, %	0.122	0.441	0.234	0.136	0.135	0.413	− 0.120	0.462	− 0.108	0.513	0.128	0.432
FBG, mg/dL	0.005	0.975	0.051	0.746	− 0.187	0.256	− 0.238	0.139	0.095	0.563	0.117	0.473
Urea, mg/dL	0.009	0.955	0.028	0.881	− 0.195	0.248	0.019	0.908	0.147	0.385	0.081	0.619
Creatinine, mg/dL	− 0.213	0.187	− 0.024	0.863	− 0.223	0.185	− 0.214	0.185	0.250	0.136	0.212	0.190
Triglycerides, mg/dL	− 0.043	0.789	0.149	0.881	− 0.179	0.276	− 0.175	0.279	0.055	0.737	0.151	0.352
Cholesterol, mg/dL	0.320	0.044	0.059	0.348	− 0.174	0.303	− 0.243	0.136	0.223	0.184	0.195	0.234
LDL-C, mg/dL	0.310	0.052	0.051	0.751	− 0.248	0.139	0.132	0.423	0.311	0.061	− 0.194	0.236
HDL-C, mg/dL	− 0.146	0.356	− 0.049	0.759	− 0.184	0.262	0.141	0.387	0.086	0.601	− 0.073	0.654
LDL to HDL, −	0.379	0.016	0.080	0.617	− 0.305	0.066	0.188	0.252	0.408	0.012	− 0.215	0.189
Insulin, μU/mL	− 0.143	0.365	0.414	0.006	− 0.209	0.202	− 0.261	0.104	0.170	0.300	0.262	0.103
WBC, ×109/L	− 0.215	0.183	− 0.153	0.335	0.081	0.634	0.004	0.980	− 0.080	0.637	0.103	0.526

*WHR *waist-to-hip ratio, *BMI* body mass index, *FBG* fasting blood glucose, *HOMA-IR* homeostasis model assessment of insulin resistance, *TG* triglycerides, *HDL-C* high-density lipoprotein cholesterol, *LDL-C* low-density lipoprotein cholesterol, *SBP* systolic blood pressure, *DBP* diastolic blood pressure, *VAI* visceral adiposity index, *WBC* white blood cells, *cIMT* carotid intima-media thickness

**Table 4:** The correlation of CTRP1 and 5 circulating levels with anthropometric characteristics and biochemical data in total study population

	CTRP 1,ng/mL		CTRP 5, ng/mL		CTRP 1/5	
	r	p	r	p	r	p
CTRP 1, ng/mL	1		0.015	0.897	0.198	0.080
CTRP 5, ng/mL	0.015	0.897	1		− 0.873	0.000
CTRP 1/5, −	0.198	0.080	− 0.873	0.000	1	
Adiponectin, μg/mL	− 0.152	0.169	− 0.121	0.289	0.161	0.157
Age, years	− 0.080	0.470	0.013	0.906	0.061	0.593
SBP, mmHg	0.027	0.806	0.009	0.934	− 0.018	0.873
DBP, mmHg	0.065	0.559	− 0.011	0.926	− 0.013	0.91
VAI, −	0.189	0.093	− 0.155	0.185	0.091	0.437
BMI, kg/m^2^	0.133	0.226	0.015	0.894	− 0.053	0.643
WHR, −	− 0.274	0.012	0.020	0.860	− 0.007	0.954
HOMA-IR, −	0.292	0.007	− 0.296	0.008	0.272	0.015
cIMT, mm	0.205	0.062	− 0.122	0.284	0.260	0.020
Visceral Fat, %	0.213	0.051	− 0.015	0.893	0.025	0.829
FBG, mg/dL	0.105	0.340	− 0.219	0.052	0.130	0.252
Urea, mg/dL	− 0.068	0.541	− 0.085	0.461	0.128	0.267
Creatinine, mg/dL	0. 169	0.129	− 0.178	0.121	0.184	0.110
Triglycerides, mg/dL	0.032	0.769	− 0.171	0.131	0.090	0.429
Cholesterol, mg/dL	0.201	0.071	− 0.199	0.085	0.212	0.066
LDL-C, mg/dL	0.183	0.101	− 0.065	0.574	0.094	0.417
HDL-C, mg/dL	− 0.103	0.352	− 0.035	0.762	0.018	0.874
LDL to HDL, −	0.166	0.137	− 0.010	0.933	0.069	0.556
Insulin, μU/mL	0.112	0.311	− 0.218	0.054	0.191	0.092
WBC, ×109/L	− 0.103	0.359	0.014	0.904	− 0.063	0.586

*WHR *waist-to-hip ratio, *BMI* body mass index, *FBG* fasting blood glucose, *HOMA-IR* homeostasis model assessment of insulin resistance, *TG* triglycerides, *HDL-C* high-density lipoprotein cholesterol, *LDL-C* low-density lipoprotein cholesterol, *SBP* systolic blood pressure, *DBP* diastolic blood pressure, *VAI* visceral adiposity index, *WBC* white blood cells, *cIMT* carotid intima-media thickness

Also, we performed multivariate stepwise linear regression analysis with cIMAT as a dependent variable and ratio of CTRP1 to CTRP5 and plasma levels of CTRP1, CTRP5, and adiponectin as independent variables. Our results showed that the ratio of CTRP1 to CTRP5 in plasma (β = 0.648, P = 0.005) and CTRP5 circulating levels (β = 0.444, P = 0.049) are significant predictors for cIMT value.

## Discussion

A grown body of evidence highlights the crucial role of adiponectin and CTRP family members in the pathogenesis of metabolic disorders [28, 29]. However, few studies have ever attempted to associate the CTRP1, CTRP5, and adiponectin circulating levels with unfavorable obesity indices including VAI, VAT, HOMA-IR, and also cIMT in T2D patients.

As for CTRP1, here, we demonstrated that circulating levels of CTRP1 were higher in T2D patients compared to those in controls, in contrast to the reduced trend of adiponectin serum levels. Moreover, binominal logistic regression analysis revealed that elevated CTRP1 plasma levels were a possible indicator of T2D. In line with these findings, previous studies reported higher plasma levels of CTRP1 in T2D and NAFLD patients in comparison with healthy participants. [29, 30]. Moreover, it was found that circulating CTRP1 in adiponectin- null mice were significantly enhanced relative to controls that confirms our finding concerning different serum patterns of CTRP1 and adiponectin [10]. However, in another study, CTRP1 levels did not significantly higher in diabetic subjects [31]. The main reason for this discrepancy could be related to the criteria used for selecting participants and obvious differences between human and animal models (DIO or *ob/ob* mice).

Interestingly, we found that CTRP1 markedly correlated with WHR and HOMA-IR and with VAI and VAT with a borderline significance. In line with our results, previous data reported that CTRP1 levels significantly increased in obese condition and associated with metabolic indices such as BMI and HOMA-IR [32–35]. Moreover, in line with current literature, Bai et al. observed higher circulating levels of CTRP1 were significantly associated with hyperglycemia and HOMA-IR in T2D patients [29]. However, another study indicated that CTRP1 circulating levels significantly decreased in diet-induced obese mice relative to normal diet mice [33, 36].

Based on discrepancies in research data regarding the different patterns of CTRP1 in the context of obesity, future studies are needed to unravel underlying mechanisms in which CTRP1 regulates energy metabolism.

Although based on the current study, we cannot explain the exact role of CTRP1 in the context of T2D, several possibilities can be deduced from other studies. For instance, the inhibition of CTRP1 impairs glucose homeostasis and insulin signaling [17, 37]. Moreover, overexpression of recombinant CTRP1 could efficiently diminish serum levels of glucose in mice, suggesting that CTRP1 may have specified overlapping roles as adiponectin [10]. This data corroborates our results regarding the differential pattern of CTRP1 and adiponectin in T2D conditions. It seems likely that the elevation of CTRP1 circulating levels in T2DM subjects has a compensatory response to the abnormal glucose and lipid metabolism. However, more clinical studies are needed to establish this concept.

There is also ample evidence about the possible role of CTRP1 in coronary artery disease and atherosclerosis [38–40]. Besides, it has been shown that the circulating level of CTRP1 is associated with coronary artery disorders and the atherosclerotic extent index. Also, the close associations of CTRP1 with an unfavorable metabolic profile can be considered as a possible reason for the relationship between CTRP1 and cardiovascular incidence risk. Here, we reported a positive correlation of CTRP1 with cIMT with a borderline significance. It has been noted that adiponectin (a paralogue of CTRP1) has potential anti-atherogenic properties and might be an independent factor correlated with atherosclerosis. Hence, it is tempting to speculate that a high level of CTRP1 is independently associated with subclinical atherosclerosis and vascular injury.

To put these findings together, our results along with others suggest that measurement of circulating CTRP1 concentrations may be valuable for assessment of cardiovascular risk. However, future researches are required to elucidate the impact of CTRP1 on cardiovascular homeostasis.

As for CTRP5, we found that CTRP5 circulating levels and CTRP1/CTRP5 ratio might be two independent associated with the cIMT index. It has been demonstrated that serum CTRP5 levels were significantly increased in patients with CAD and positively correlated with the extent and severity of atherosclerosis in these patients [47].

Recently, CTRP5 has been found as a mediator of metabolic pathways involved in T2D, insulin resistance, inflammation, and also obesity-related cardiovascular abnormalities[41]. In the present study, circulating CTRP5 concentrations were lower in T2D patients with a borderline significant level. Nevertheless, the previous results are inconsistent. There is a report about the low levels of circulating CTRP5 in T2D subjects, whereas another study argued that circulating levels of CTRP5 were significantly higher in obese and diabetic mice rather than lean groups [41, 42]. An in vivo study by Lei et al. revealed that CTRP5 circulating levels were not significantly changed in ob/ob mice [43]. Besides, we previously showed that plasma CTRP5 levels were significantly lower in NAFLD and T2D patients in comparison with healthy subjects [44]. As mentioned above, there is a discrepancy between animal model studies and human surveys that are might be due to the different functions of CTRP5 in humans and mice; just as resistin plays different roles in humans and mice[45]. Another reason might be due to different genetic background that affects phenotype since T2D in humans is a heterogeneous disease which is associated with environmental factors, whereas ob/ob and db/db mice are only caused by genetic manipulation or high-fat diet [46]. Also, it is noteworthy that we observed a negative correlation between CTRP5 and HOMA-IR.In parallel, inhibition of CTRP5 action can alleviate insulin resistance associated with obesity and diabetes [43]. However, there is a limited number of data on the correlation between CTRP5 and type 2 diabetes in humans and future clinical studies are demanded.

Taken together, as a preliminary study, our study indicates that the CTRP1 to CTRP5 ratio in plasma may be associated with cIMT among men with T2D. However, more studies in large sample sizes are required to clarify the role of CTRPs in the pathomechanism of T2D and associated metabolic abnormalities. It is also worth mentioning that the underlying mechanism of association between CTRP1 and CTRP5 alterations and metabolic abnormalities cannot be elucidated based on the current study. Although our results accompanying available literature can provide novel information on the role of CTRP1 and CTRP5 in the pathomechanism of metabolic disorders, several limitations of the study should be considered.

The main limitation is the small number of patients enrolled in this study which precluded any definitive conclusions on the exact role of CTRP1 and CTRP5 in the context of T2D, and cardiometabolic risk factors. To keep gender homogeneity, our study as a preliminary report was restricted to men who represent the vast majority of patients with T2D. However, gender difference seems to affect the CTRP1 and CTRP5 levels. Therefore, future study with a larger sample size in both men and women is necessary to establish the role of CTRPs in the pathogenesis of T2D. Moreover, the gender difference in cIMT was reported in several studies [48, 49]. Hence, there is a need for future work to determine if the CTRP1/CTRP5 ratio in women is also a predictor of cIMT.

## Conclusions

In summary, it seems that the enhancement of CTRP1 levels along with reduced CTRP5 circulating levels was associated with an increase in the risk of T2D in men. Moreover, the CTRP1 to CTRP5 ratio in plasma may be associated with cIMT among T2D patients. However, more investigations with a larger sample size in both sex are needed to confirm this concept.

## Data Availability

The datasets used and analyzed during the current study are available from the corresponding author on reasonable request.

## Abbreviations

CTRP: C1qTNF-related protein
T2D: Type 2 Diabetes
VAT: Visceral adipose tissue
SAT: Subcutaneous adipose tissue
VAI: Visceral adiposity index
cIMT: Carotid intima-media thickness
ELISA: Enzyme-linked immunosorbent assay
CVD: Cardiovascular disease
ACC: Acetyl-CoA carboxylase
BMI: Body mass index
WHR: Waist-to-hip ratio
TG: Triglycerides
HDL-C: High-density lipoprotein cholesterol
LDL-C: Low-density lipoprotein cholesterol
AST: Aspartate aminotransferase
ALT: Alanine aminotransferase
ALP: Alkaline phosphatase
γ-GT: Gamma-glutamyl transferase
HOMA-IR: Homeostasis model assessment for insulin resistance
OGTT: Oral glucose tolerance test
